# Development, Implementation, and Evaluation of a Telemedicine Service for the Treatment of Acute Stroke Patients: TeleStroke

**DOI:** 10.2196/ijmr.2163

**Published:** 2012-11-15

**Authors:** Carlos Parra, Francisco Jódar-Sánchez, M. Dolores Jiménez-Hernández, Eduardo Vigil, Alfredo Palomino-García, Francisco Moniche-Álvarez, Francisco Javier De la Torre-Laviana, Patricia Bonachela, Francisco José Fernández, Aurelio Cayuela-Domínguez, Sandra Leal

**Affiliations:** 1 Virgen del Rocío University HospitalTechnological Innovation GroupSevilleSpain; 2 Virgen del Rocío University HospitalNeurological DepartmentSevilleSpain; 3 Virgen del Rocío University HospitalClinical Documentation ServiceSevilleSpain; 4 Virgen del Rocío University HospitalResearch, Development and Innovation DepartmentSevilleSpain; 5 EverisResearch and Technological Development GroupSevilleSpain; 6 Seville DistrictSevilleSpain

**Keywords:** Telemedicine, Standardization, Stroke, Fibrinolysis

## Abstract

**Background:**

Health care service based on telemedicine can reduce both physical and time barriers in stroke treatments. Moreover, this service connects centers specializing in stroke treatment with other centers and practitioners, thereby increasing accessibility to neurological specialist care and fibrinolytic treatment.

**Objective:**

Development, implementation, and evaluation of a care service for the treatment of acute stroke patients based on telemedicine (TeleStroke) at Virgen del Rocío University Hospital.

**Methods:**

The evaluation phase, conducted from October 2008 to January 2011, involved patients who presented acute stroke symptoms confirmed by the emergency physician; they were examined using TeleStroke in two hospitals, at a distance of 16 and 110 kilometers from Virgen del Rocío University Hospital. We analyzed the number of interconsultation sheets, the percentage of patients treated with fibrinolysis, and the number of times they were treated. To evaluate medical professionals’ acceptance of the TeleStroke system, we developed a web-based questionnaire using a Technology Acceptance Model.

**Results:**

A total of 28 patients were evaluated through the interconsultation sheet. Out of 28 patients, 19 (68%) received fibrinolytic treatment. The most common reasons for not treating with fibrinolysis included: clinical criteria in six out of nine patients (66%) and beyond the time window in three out of nine patients (33%). The mean “onset-to-hospital” time was 69 minutes, the mean time from admission to CT image was 33 minutes, the mean “door-to-needle” time was 82 minutes, and the mean “onset-to-needle” time was 150 minutes. Out of 61 medical professionals, 34 (56%) completed a questionnaire to evaluate the acceptability of the TeleStroke system. The mean values for each item were over 6.50, indicating that respondents positively evaluated each item. This survey was assessed using the Cronbach alpha test to determine the reliability of the questionnaire and the results obtained, giving a value of 0.97.

**Conclusions:**

The implementation of TeleStroke has made it possible for patients in the acute phase of stroke to receive effective treatment, something that was previously impossible because of the time required to transfer them to referral hospitals.

## Introduction

The World Health Organization predicts that by 2030 cerebrovascular disease will be the second leading cause of death in the world; the percentage of deaths due to this disease will increase from 9.7% in 2004 to 12.1% in 2030 [1]. In Andalusia in 2007, cerebrovascular diseases presented a crude mortality rate of 86 per 100,000 inhabitants, 12% higher than that for Spain as a whole [2]. It has been demonstrated that both mortality and disability are reduced when these patients are treated by teams specially trained in administering treatment for this type of disease [3]. Therefore, specialized teams are required at referral hospitals (RH) that can respond to consultations from other hospitals without specialist neurologists. Interhospital networks based on urgent transfer have been shown to only partially ensure geographical equity in access to quality medical interventions at the cost of many unnecessary transfers [4].

Health care service based on telemedicine can reduce both physical and time barriers in stroke treatments. Moreover, this service connects centers specializing in stroke treatment with other centers and practitioners, thereby increasing accessibility to neurological specialist care and fibrinolytic treatment.

The principal competitive advantage of the telemedicine system over other similar solutions is its use of communications standards, providing the system with greater flexibility and interoperability [5]. Another advantage of the system is that its development has been directed towards the care process, and expert health personnel have been involved in the design and definition of requirements.

Previous studies used real-time video conference systems for acute stroke care to evaluate clinical neurological status with the National Institute of Health Stroke Scale (NIHSS) [6-8] and computerized tomography (CT) images [9-16]. The evolution of this technology has led telemedicine to be considered as an essential method for the diagnosis and treatment of patients suspected of having had a stroke. Communications via Internet and mobile phone may be the best means of access for all doctors treating these patients without requiring the patients’ transfer [17]. This method of work is clearly beneficial to patients, their environment, and health institutions [18]. It has been shown that acute phase treatments can be applied without having to travel long distances and in the absence of resources and high-cost complex structures, which are probably not available at all points of care for these patients [19]. Consequently, it is possible for a team of stroke experts to be available 24 hours a day, at any hospital, and at relatively low cost [20].

### Previous Experience in Spain

There are two studies on previous telemedicine applications for the treatment of stroke patients in Spain at the Vall d’Hebron University Hospital [11] in Barcelona and Son Espases University Hospital [16] in Palma de Mallorca. However, these experiences did not include interoperability capabilities with the Electronic Health Record, which is a central issue for the continuity of care [21].

### Current Study

This paper describes the development, implementation, and evaluation of a care service based on telemedicine for the emergency treatment of patients with acute stroke at Virgen del Rocío University Hospital in Seville, a fully equipped stroke center.

## Methods

### Development

The development of the project involves a health care process modeling and development system.

#### TeleStroke Process Model

Business process modeling (BPM) is a methodology that has been applied before in this area [22]. In fact, it has been used in the health care sphere for years, particularly in the field of information system design. Use of this methodology in health care processes continues to become increasingly common [23-27].

In order to model the treatment process for TeleStroke, we chose business process modeling notation (BPMN), which provides a standard language permitting business process modeling in a workflow format. Its main goal is to provide a standard notation that will be easily read and understood by everyone involved in and affected by the business (ie, the stakeholders). These stakeholders include the users themselves who carry out the process, business analysts who define and redefine processes, technical developers responsible for implementing processes, and business managers and administrators who monitor and direct processes [28].

Modeling began with an exhaustive study of the documentation related to this condition and the contributions from the group of stroke specialists. Subsequently, this information was processed and made into a BPM model using BPMN.

#### TeleStroke System

The system has been designed to meet the specifications of the World Health Organisation program, eHealth for Health Care Delivery (eHCD). The eHCD states that eHealth technologies must be adapted and applicable to the different health systems [29] in accordance with their possibilities and resources, directed towards specific objectives, complying with international standards and in an established manner. In this way, the international standard Health Level Seven (HL7) [30] is used for the format of data and exchange of information and the protocol, digital imaging, and communication in medicine (DICOM) [31] as the internationally recognized standard for the exchange of medical images. Following the recommendations of the eHCD, we have drawn on the experience of specialist personnel at the RH to decide which functions must be supported by the system to enable the diagnosis and treatment of stroke patients.

The interoperability scenario is supposed to enable the automation of communication, making the system responsible for collecting patient data and information, medical records, admission details, laboratory data, and images from the picture archiving and communication systems (PACS), etc., to be sent to the RH. One essential and critical requirement in the interoperability of the systems is the traceability of information.

The stages of the interoperability scenarios involved in TeleStroke are as follows:

a) Stage 1: Requesting hospital. The patient arrives at the requesting hospital, normally through the emergency department. The patient is examined at the hospital, suspected as a stroke case, and as a result, the doctor calls the TeleStroke Medical Station.

b) Stage 2: Interconsultation request. The emergency doctor submits an interconsultation request from the TeleStroke Medical Station to the RH. This, in turn, becomes a notification of incoming interconsultation to the mobile phone of the on-call neurologist, who goes to the medical station to collect the patient’s data and accept the interconsultation.

c) Stage 3: TeleStroke interconsultation. Once the interconsultation has started, the neurologist may ask for images via videoconference, request further details about the patient, or send instructions to be carried out in the examination of the patient.

d) Stage 4: Closure and record. On conclusion of the examination, the specialist closes the interconsultation, providing details of diagnosis and treatment, so that the doctor at the requesting hospital can complete the information required in the Stroke Record System. The system architecture consists of two different elements: the TeleStroke Medical Station and communication between medical stations.

### Implementation of Platform

The RH is the Virgen del Rocío University Hospital, which has an Electronic Health Record, called SIDCA, enabling the management of information and knowledge. Two hospitals were selected as requesting hospitals: San Juan de Dios (SJDH) and José María Díaz Domínguez (JMDDH). Work on the development of the platform began with a study of the infrastructure existing in the participating centers responsible for consultation with the RH. Table 1 shows the features of the information systems installed in both hospitals.

The system is based fundamentally on communication between the requesting hospital where the patient is located and the RH where the specialist group is based.

**Table 1:** Information systems at the two requesting hospitals.

Features	San Juan de Dios Hospital	José María Díaz Domínguez Hospital
HIS^a^	HIS ACTICX Telvent Interactive 4.1. HL7 communication. HL7 communication.	Aurora from SIEMENS, version 3.0.78. HL7 communication. HL7 communication.
LIS^b^	OpenLAB from Icon Media Lab. HL7 communication. HL7 communication.	Omega 2000 from ROCHE version 2.02.00.b. HL7 communication. HL7 communication.
RIS^c^	PHILIPS. HL7 communication. HL7 communication.	Telvent GESIR 3.0. HL7 communication.
PACS^d^	PACS from PHILLIPS. DICOM communication. DICOM communication.	PACS from Telvent. DICOM communication. DICOM communication.
Security and authentication	Firewall. They currently have an Active Directory	Firewall. Rt domain.
Communications	Symmetric communication with Corporate Network through MACROLAN, 2 Mbps	Symmetric communication with Corporate Network via 100Mb Optical Fibre MetroLAN and 2MB Reinforced Copper MetroLan

^a ^Hospital Information System.

^b ^Laboratory Information System.

^c ^Radiology Information System.

^d ^Picture Archiving and Communication Systems.

### Evaluation

#### Study Design

This prospective observational study was conducted from October 2008 to January 2011 with patients who presented acute stroke symptoms. Patient inclusion criteria were: less than 4.5 hours after onset of stroke, age between 18 and 80 years (individualized decision in patients over 80 years according to baseline), and NIHSS scale between 5 and 24. They were examined using TeleStroke in SJDH and JMDDH, which are 16 and 110 kilometers respectively from Virgen del Rocío University Hospital. These hospitals did not have a fully equipped stroke center and patients with acute stroke symptoms could not be treated with fibrinolysis before the introduction of the TeleStroke system.

The Ethics Committee at Virgen del Rocío University Hospital approved this study.

#### Outcome Measures

We analyzed the number of interconsultation sheets, the percentage of patients treated with fibrinolysis, the time from onset of stroke symptoms to admission (“onset-to-hospital”), time from admission to fibrinolysis (“door-to needle”), time from admission to CT image, and time from onset of stroke symptoms to fibrinolysis (“onset-to-needle”). We also analyzed the reason why patients were not treated with fibrinolysis.

To evaluate medical professionals’ acceptance of the TeleStroke system, we developed a web-based questionnaire using a Technology Acceptance Model (TAM) [32]. The TAM is a model based on the intended use of new technologies; it was developed to explain and predict the acceptance of information technology by potential users. The methodology has been used in other telemedicine studies [33].

The original TAM model included two dimensions to determine intended use: perceived usefulness and ease of use. For our study, we extended the original model by including two more dimensions: social norms and enabling conditions. Thus, in the end we evaluated five dimensions. Items used in the questionnaire were chosen from the literature [34]. The validity of the questionnaire’s content was evaluated by a panel of experts consisting of doctors and experts in health technology assessment. Some of the questionnaire items were modified after the review of the experts. Under these conditions, we developed the questionnaire in a web form with 27 questions whose items were sorted randomly to avoid bias. In addition to the scores for questions, we used a ten-point Likert scale: one point for strongly disagree and ten points for totally agree. Once we had created the questionnaire, it was emailed to neurologists at Virgen del Rocío University Hospital, and family physicians, internists, and intensivists at SJDH and JMDDH.

#### Data Analysis

Descriptive analysis of data used absolute (n) and relative (%) frequencies for qualitative variables and mean ± standard deviation (SD) for quantitative variables. We measured the reliability of the instrument by calculating Cronbach alpha.

## Results

### Development

#### TeleStroke Process Model

The process model is universally considered to be the first step in analyzing and improving processes. It defines a model that contains all relevant characteristics of the actual process and makes analyzing that process possible. The process modeling was carried out based on acute-phase stroke treatment guides and protocols and the experience of specialists working with this condition (see Multimedia Appendix 1).

#### TeleStroke System

**Figure 1:**
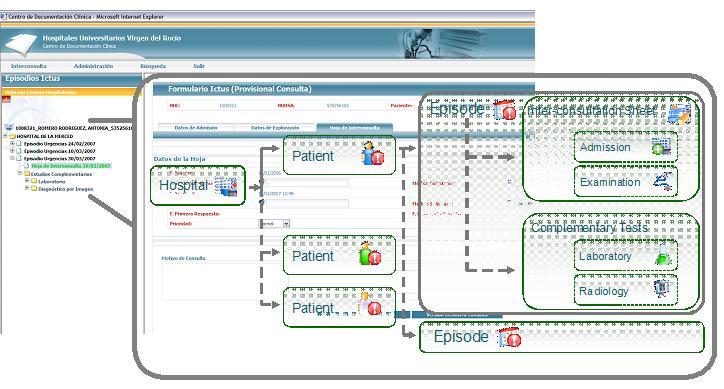
Data hierarchy from TeleStroke medical station.

The TeleStroke Medical Station is responsible for collecting the necessary information from the departmental applications (Hospital Information System, Radiology Information System, Laboratory Information System and PACS) and sending it to the RH. In accordance with the HL7 standard, the information is collected using batch and online procedures. The user interface in the TeleStroke Medical Station has the emergency episode (Figure 1) as a central element.

The user interface has the following information items:

1) The interconsultation sheet. Through this sheet the doctor determines when the health personnel at the requesting hospital may consult the RH. It includes the information required by the specialist group to confirm diagnoses. It includes the following elements:

- Examination details: information about the “in situ” examination of the patient. This includes vital signs (heart rate, body temperature, etc.), personal history (stroke, heart disease, hypertension, etc.), inclusion and exclusion criteria, scales (NIHSS), complementary tests (hemogram, electrocardiogram, etc.), and complementary details.
- Admission details: details of patient’s admission to hospital.
- Consultation details: record of the consultation with the RH, including replies, assessments, and diagnoses of the group of specialists at the RH.

2) Complementary data consist of the following:

- Laboratory data: study details (request, date, etc.) and test results, organized by section, chapter, and test.
- Radiology data: study details (request, date, etc.) and examination (date, results, etc.) together with CT images.

3) Navigation tree is based on the data hierarchy from the medical station.

### Implementation

Communication between requesting hospitals and the RH is via Andalusia regional government’s Corporate Network, which has been certified as a safe network. The client equipment that serves as TeleStroke Medical Station is located in a reserved area in the requesting hospital. At this point, we need to highlight the three different elements in communications between medical stations:

1) Transfer of episode and interconsultation sheet. The interconsultation sheet shared between both hospitals has 4 possible statuses:

- Provisional consultation: the user of the TeleStroke Medical Station saves details of the patient’s examination but has not yet consulted the specialist neurologist group.
- Definitive consultation: details and consultation are sent to RH (SIDCA).
- Provisional reply: the user of the medical station at SIDCA has saved a reply to the consultation but has not yet sent it to the requesting hospital.
- Definitive reply: the reply is sent to the requesting hospital.

During the lifecycle of the interconsultation sheet and its complementary data, data modifications may arise that must be shared with the requesting hospital and the RH.

**Figure 2:**
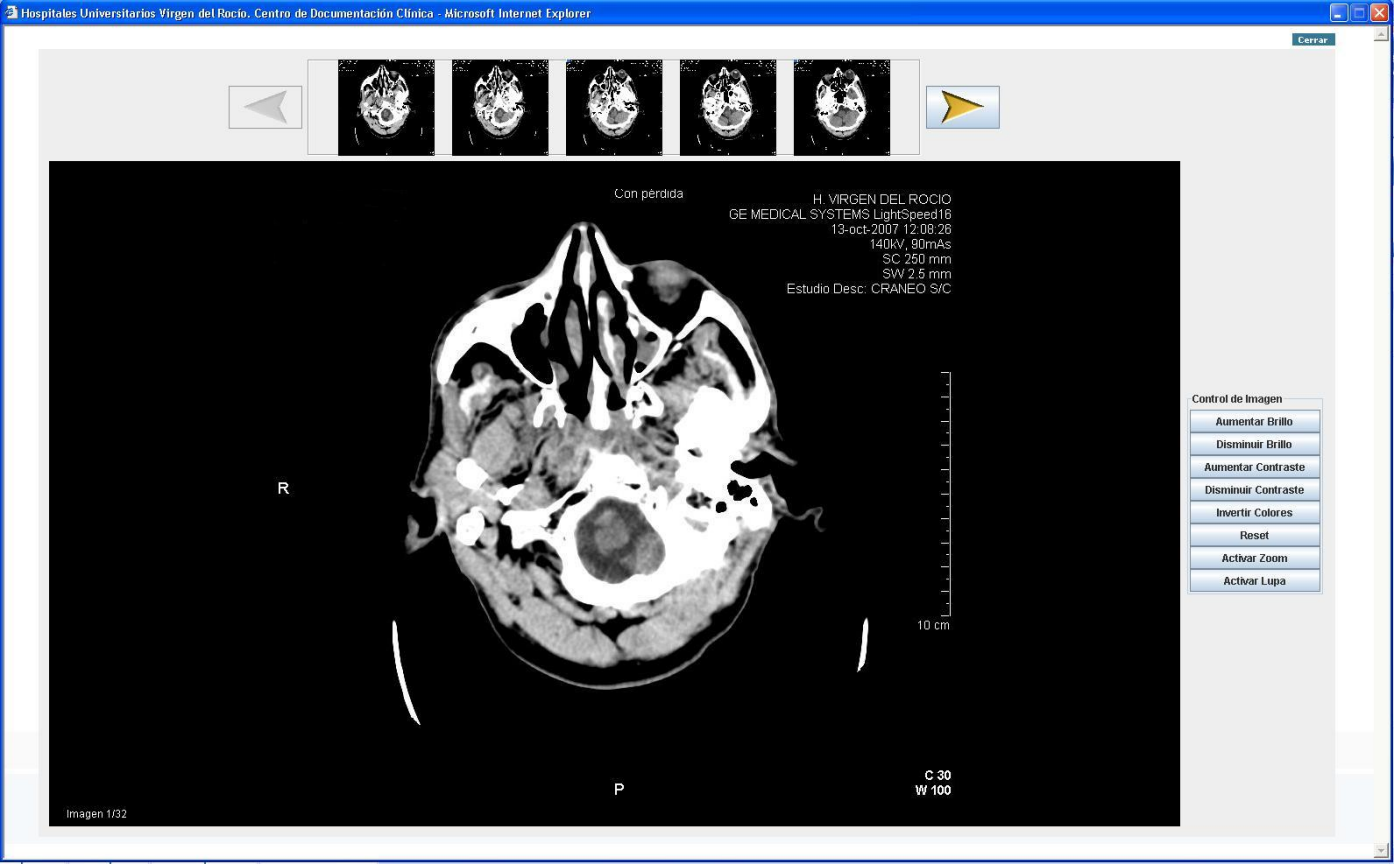
Computerized tomography image.

2) Processing, sending and displaying images. Images are collected from DICOM servers. Given the basic requirement of speed in image transmission in order to obtain an urgent diagnosis, images are compressed in accordance with the general-purpose image compression standard. JPEG [35] is also used to compress images automatically. The DICOM images enable a series of display functions that are used by health personnel and have also been developed for JPEG images. Figure 2 shows the CT image display including both the TeleStroke Medical Station and the Medical Station at the RH for displaying PACS images compressed using the JPEG standard. All image processing is performed in the web application itself without requiring installation of additional components in the client equipment.

3) Videoconference and chat. This application allows compartmentalization of the browser window, remote desktop, or blackboard application. It also permits sending files and recordings of videos or sessions.

### Evaluation

A total of 28 patients, 19 from SJDH and 9 from JMDDH, were evaluated through interconsultation sheets. Table 2 summarizes the main characteristics of patients.

Out of 28 patients, 19 (68%) received fibrinolytic treatment. These 19 patients were those who met the inclusion criteria. We analyzed four process indicators for fibrinolysis delivery (Table 3). The most common reasons for not treating with fibrinolysis were: clinical criteria in six out of nine patients (66%) and beyond the time window in three out of nine patients (33%).

Table 4 summarizes the main characteristics of the medical professionals who completed the questionnaire on acceptance of Telestroke. In total, we sent out 61 questionnaires, of which we received 34 (56%) completed. Table 5 shows the descriptive statistics analysis of the items questionnaire. As shown, the mean values for each item were over 6.50, indicating that respondents positively evaluated each item. To assess the reliability of the questionnaire we calculated the Cronbach alpha value, obtaining a value of 0.97.

**Table 2:** Characteristics of patients; data are presented as n (%).

		No Fibrinolysis	Fibrinolysis
Age	40 – 49	2 (22.2)	3 (15.8)
	50 – 59	2 (22.2)	1 (5.0)
	60 – 69	1 (11.1)	4 (21.0)
	70 – 80	4 (44.4)	11 (58.2)
Sex	Females	3 (33.3)	10 (52.6)
	Males	6 (66.7)	9 (47.4)
Origin^a^	Own decision	3 (33.3)	6 (35.3)
	Health center	3 (33.3)	4 (23.5)
	Emergency department	0 (0)	1 (5.9)
	MICU^b^	3 (33.3)	7 (35.3)
Displacement of the patient	Own vehicle	4 (44.4)	7 (36.8)
	Ambulance	5 (55.6)	12 (63.2)

^a ^There are values missing for these variables.

^b ^MICU: Mobile Intensive-Care Unit.

**Table 3:** Indicators in process of fibrinolysis delivery.

		Min-Max	Mean ± SD
**Time**			
	Onset-to-hospital	15-155	68.68 ± 41.76
	Door-to-needle	33-128	81.89 ± 28.29
	Admission-to-CT scan	6-85	32.87 ± 19.11
	Onset-to-needle	70-210	150.58 ± 38.61

**Table 4:** Characteristics of medical professionals who have used TeleStroke; data are presented as n (%).

Categories		SJDH	JMDDH	VRUH	Total
Sex	Females	6 (17.6)	1 (2.9)	6 (17.6)	13 (38.2)
	Males	13 (38.2)	3 (8.8)	5 (14.7)	21 (61.8)
Age	<40	9 (26.4)	1 (2.9)	8 (23.5)	18 (52.9)
	40-49	9 (26.4)	2 (5.9)	2 (5.9)	13 (38.2)
	≥50	1 (2.9)	1 (2.9)	1 (2.9)	3 (8.8)
Clinical practice years	<10	2 (5.9)	-	5 (14.7)	7 (20.6)
	10-19	17 (50.0)	3 (8.8)	4 (11.7)	24 (70.6)
	≥20	-	1 (2.9)	2 (5.9)	3 (8.8)
Medical speciality	Family doctors	7 (20.6)	-	-	7 (20.6)
	Internists	9 (26.5)	-	-	9 (26.5)
	Intensivists	3 (8.8)	4 (11.7)	-	7 (20.6)
	Neurologists	-	-	11 (32.3)	11 (32.3)
Experience with TeleStroke	Knowledge/training courses	6 (17.6)	-	2 (5.9)	8 (23.5)
	Clinical case	13 (38.2)	4 (11.7)	9 (26.5)	26 (76.5)

**Table 5:** Descriptive statistics of the TAM questionnaire item.

Items		Min-Max	Mean ± SD
**Perceived usefulness**			
	TS could enhance my effectiveness of job	1-10	7.35 ± 2.06
	TS would allow greater control over DTP^a^	1-10	7.68 ± 1.98
	TS could support critical aspects in DTP	3-10	8.18 ± 1.80
	If I use TS^b^, I will increase my chances to develop my career	1-10	7.06 ± 2.36
	Using TS would make my job easier	1-10	6.62 ± 2.62
	Using TS would improve my job performance	1-10	7.32 ± 2.03
	Using TS would help me to accomplish DTP more quickly	1-10	6.97 ± 2.89
	TS could improve the quality of DTP that I deliver	3-10	7.82 ± 1.87
	Overall, TS could be useful to improve DTP	1-10	8.06 ± 1.98
**Perceived ease of use**			
	Learning to use TS would be easy for me	4-10	7.56 ± 1.71
	My interaction with TS would be clear and understandable	3-10	7.09 ± 1.91
	I think that DTP made through TS would be clear	3-10	7.82 ± 1.80
	It would be easy for me to become skillful at using TS	4-10	7.21 ± 1.74
	Overall, I believe that TS will be easy to use	2-10	6.65 ± 2.14
**Subjective norm**			
	Colleagues whose opinions I value think I should use TS	3-10	7.91 ± 1.91
	My superiors think that I should use TS	8-10	9.47 ± 0.75
	Other health professionals whose opinions I value think I should use TS	3-10	7.91 ± 1.90
	The management of the hospital supports me to use TS	2-10	9.03 ± 1.66
	Overall, I believe that the hospital supports the use of TS	5-10	8.94 ± 1.32
**Facilitating conditions**			
	A specific person will be available to solve problems regarding to TS	1-10	6.65 ± 2.00
	I will have the resources necessary to use TS	3-10	7.94 ± 1.84
	I will receive training to use TS	2-10	7.12 ± 2.21
	TS will be compatible with other systems I use	4-10	8.18 ± 1.58
	The hospital has the infrastructure necessary to I use TS	4-10	7.91 ± 1.83
**Intention to use**			
	I intend to use TS as it is available in the hospital	4-10	9.03 ± 1.55
	I intend to use TS for DTP as often as needed	4-10	8.97 ± 1.42
	Whenever possible, I intend to use TS	3-10	8.82 ± 1.73

^a ^TS: TeleStroke.

^b ^DTP: diagnosis and treatment of patients.

## Discussion

The implementation of TeleStroke has made it possible for patients in the acute phase of stroke to receive effective treatment, something that was previously impossible because of the time required to transfer them to the RH. In 2006, before implementing the TeleStroke service, 57% of acute stroke patients transferred to the Virgen del Rocío University Hospital emergency department from another hospital presented more than 4.5 hours after the onset of symptoms [36]. However, it is important to note that patients who arrived at the Virgen del Rocío University Hospital emergency department between 3 and 4.5 hours after the onset of symptoms may not be treated with fibrinolysis because of the time required for performing diagnostic tests. Given that this situation does not satisfy the recommended maximum time for treatment, it justifies the deployment of the telemedicine service.

System reliability is vital for the successful implementation of a telemedicine system with a high impact on care. In this sense, formal and rigorous methods of risk management are required, as is good capacity management of the technology to guarantee the service level. We demonstrated that reliability was also essential, and we subjected the system to an exhaustive testing process to ensure this.

Several technological upgrades were carried out during the period of the study in order to increase the system usability and reliability, improving the medical staff access and therefore decreasing the “door-to-needle” time. In turn, the application of BPM modeling techniques to model the care protocol proved to be the right choice because it led to explicit agreement between health professionals on the best way to work. This will make it easier to export it to the rest of the Andalusian Public Health System.

The need to accelerate the communication time of the CT images to the RH has been made clear. Despite conversion to JPG format greatly reducing file size, this remains the most important time period in the whole process. To this end, we have checked the diagnostic validity of JPEG format CT images with regards to DICOM format CT images in a complementary study that is awaiting publication. We found a far higher percentage of patients who had received fibrinolysis compared to other studies that examined stroke patients through videoconference [9,12-14,16], while the number of patients surveyed was higher in most of these studies. The application of BPM modeling techniques may contribute to a higher fibrinolytic treatment rate facilitating agreement and training among professionals involved in the care process.

The CT image is the critical test for the successful application of fibrinolysis to patients. The mean time from admission to CT image was 33 minutes, higher than other studies [16]. In our study, one patient had a symptomatic intracranial haemorrhage detected in the CT image. The mean “onset-to-hospital” time was 69 minutes. Similar studies [9,12-14,16] reported this time ranged from 36 to 84 minutes. The mean “door-to-needle” time was 82 minutes. Similar studies [9,11-12,16] reported this time ranged from 53 to 106 minutes. The mean “onset-to-needle” time was 150 minutes. Similar studies [9-12,14,16] reported this time ranged from 122 to 157 minutes.

There were no technical failures preventing a clinical response being given to the patient within the established limits, although there were delays in receiving the CT image and problems with sound and images from the videoconferences that increased response times.

The acceptance of the TeleStroke system by health care professionals is essential for the incorporation of eServices to be successful. To assess the reliability of the questionnaire, we obtained a value of 0.97 for Cronbach alpha, higher than the recommended value of 0.7. Results of the TAM questionnaire indicate a very positive perception of questionnaire items, particularly the Intention to Use and Subjective Norms dimensions.

The authors have identified two important limitations in this study: the small sample size and the lack of patient outcomes related to mortality and functional status. These issues will be covered in a future study.

As current evidence is limited, a cost-effectiveness analysis may be required into the economic impact of telemedicine in acute stroke treatment [37] following the methodology applied in a recent study [38]. Currently, we are working on adapting the TeleStroke system to be deployed in the rest of the Andalusian Public Health System. A new line of work is the development of mobility technology to capture and transmit videoconference and CT data to allow swifter diagnosis and treatment of fibrinolysis in acute stroke patients [39].

Taking all of these discussion factors into account, we can state that the implementation of a telemedicine service for the treatment of acute stroke patients at Virgen del Rocío University Hospital is feasible, and it has been proven that TeleStroke facilitates the accessibility to neurological specialist care and fibrinolytic treatment in hospitals where a fully equipped stroke center is not available.

## Multimedia Appendix 1

Process model TeleStroke.

## Abbreviations

BPM: Business Process Modeling
BPMN: Business Process Modeling Notation
CT: Computerized Tomography
DICOM: Digital Imaging and Communication in Medicine
eHCD: eHealth for Health Care Delivery
HL7: Health Level Seven
JMDDH: José María Díaz Domínguez Hospital
NIHSS: National Institute of Health Stroke Scale
RH: Referral Hospital
SJDH: San Juan de Dios Hospital
SD: Standard Deviation
TAM: Technology Acceptance Model
