# Supplementary figures and images for: Development, Implementation, and Evaluation of a Telemedicine Service for the Treatment of Acute Stroke Patients: TeleStroke
Source: Interact J Med Res. 2012 Nov 15;1(2):e15. doi: 10.2196/ijmr.2163 (PMC3626126; doi:10.2196/ijmr.2163)

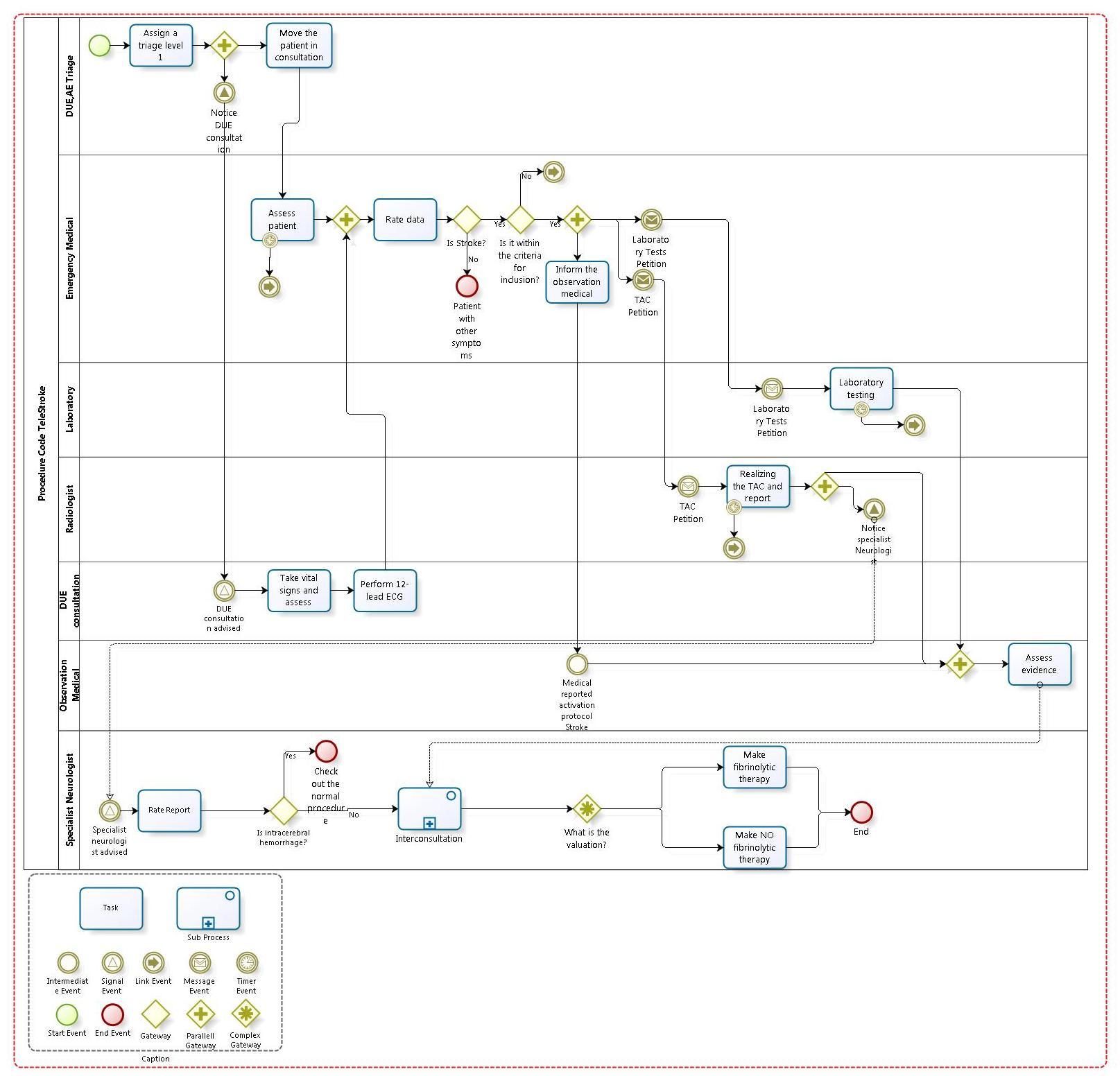

Supplement: Supplementary file 1 [file ijmr_v1i2e15_app1.JPG]
